# Oncologic Outcomes of Lymph Node Dissection at Salvage Radical Prostatectomy

**DOI:** 10.3390/cancers15123123

**Published:** 2023-06-09

**Authors:** Felix Preisser, Reha-Baris Incesu, Pawel Rajwa, Marcin Chlosta, Mohamed Ahmed, Andre Luis Abreu, Giovanni Cacciamani, Luis Ribeiro, Alexander Kretschmer, Thilo Westhofen, Joseph A. Smith, Markus Graefen, Giorgio Calleris, Yannic Raskin, Paolo Gontero, Steven Joniau, Rafael Sanchez-Salas, Shahrokh F. Shariat, Inderbir Gill, Robert Jeffrey Karnes, Paul Cathcart, Henk Van Der Poel, Giancarlo Marra, Derya Tilki

**Affiliations:** 1 Martini-Klinik Prostate Cancer Center, University Hospital Hamburg Eppendorf, 20251 Hamburg, Germany; f.preisser@uke.de (F.P.);; 2 Department of Urology, Medical University of Vienna, 1090 Vienna, Austria; 3 Department of Urology, Medical University of Silesia, 40-055 Zabrze, Poland; 4 Department of Urology, Mayo Clinic, Rochester, MN 55902, USA; 5 Keck Medical Center of USC, USC Institute of Urology, University of Southern California, Los Angeles, CA 90033, USA; 6 Urology Centre, Guy’s Hospital, London SE1 9RT, UK; 7 Department of Urology, Ludwig-Maximilians University of Munich, 80539 Munich, Germany; 8 Department of Urologic Surgery, Vanderbilt University Medical Center, Nashville, TN 37232, USA; 9 Department of Surgical Sciences, San Giovanni Battista Hospital, University of Turin, 10124 Turin, Italy; 10 Department of Urology, University Hospitals Leuven, 3000 Leuven, Belgium; 11 Department of Urology, Institut Mutualiste Montsouris, Université Paris Descartes, 75270 Paris, France; 12 Department of Urology, Second Faculty of Medicine, Charles University, 116 36 Prague, Czech Republic; 13 Hourani Center for Applied Scientific Research, Al-Ahliyya Amman University, Amman 19628, Jordan; 14 Department of Urology, Weill Cornell Medical College, New York, NY 10075, USA; 15 Department of Urology, University of Texas Southwestern, Dallas, TX 75390, USA; 16 Department of Urology, Netherlands Cancer Institute, 1066 Amsterdam, The Netherlands; 17 Department of Urology, University Hospital Hamburg-Eppendorf, 20251 Hamburg, Germany; 18 Department of Urology, Koc University Hospital, 34010 Istanbul, Turkey

**Keywords:** salvage prostatectomy, BCR, lymph node dissection, lymph node invasion, oncological outcomes

## Abstract

**Simple Summary:**

Lymph node invasion represents a poor prognostic factor after primary radical prostatectomy for prostate cancer. However, its impact on oncologic outcomes in salvage radical prostatectomy patients is unknown. Within this study we investigated the impact of lymph node invasion and dissection on the oncologic outcomes after salvage prostatectomy. Our results show that lymph node invasion represents are poor prognostic factor after salvage prostatectomy. Conversely, we recorded no benefit for lymph node dissection compared to no lymph node dissection during salvage prostatectomy. These findings underline the need for a cautious indication of lymph node dissection in salvage prostatectomy patients as well as strict postoperative monitoring of patients with lymph node invasion.

**Abstract:**

Background: Lymph node invasion (LNI) represents a poor prognostic factor after primary radical prostatectomy (RP) for prostate cancer (PCa). However, the impact of LNI on oncologic outcomes in salvage radical prostatectomy (SRP) patients is unknown. Objective: To investigate the impact of lymph node dissection (LND) and pathological lymph node status (pNX vs. pN0 vs. pN1) on long-term oncologic outcomes of SRP patients. Patients and methods: Patients who underwent SRP for recurrent PCa between 2000 and 2021 were identified from 12 high-volume centers. Kaplan–Meier analyses and multivariable Cox regression models were used. Endpoints were biochemical recurrence (BCR), overall survival (OS), and cancer-specific survival (CSS). Results: Of 853 SRP patients, 87% (*n* = 727) underwent LND, and 21% (*n* = 151) harbored LNI. The median follow-up was 27 months. The mean number of removed lymph nodes was 13 in the LND cohort. At 72 months after SRP, BCR-free survival was 54% vs. 47% vs. 7.2% for patients with pNX vs. pN0 vs. pN1 (*p* < 0.001), respectively. At 120 months after SRP, OS rates were 89% vs. 81% vs. 41% (*p* < 0.001), and CSS rates were 94% vs. 96% vs. 82% (*p* = 0.02) for patients with pNX vs. pN0 vs. pN1, respectively. In multivariable Cox regression analyses, pN1 status was independently associated with BCR (HR: 1.77, *p* < 0.001) and death (HR: 2.89, *p* < 0.001). Conclusions: In SRP patients, LNI represents an independent poor prognostic factor. However, the oncologic benefit of LND in SRP remains debatable. These findings underline the need for a cautious LND indication in SRP patients as well as strict postoperative monitoring of SRP patients with LNI.

## 1. Introduction

Salvage radical prostatectomy (SRP) is an accepted treatment modality for radio-recurrent prostate cancer. The effect of lymph node invasion (LNI) after primary radical prostatectomy (RP) is well established. Specifically, LNI is known to be a poor prognostic factor after primary RP [1]. However, it is unknown how LNI affects oncologic outcomes after SRP. Similarly, the effect of lymph node dissection (LND) at SRP on oncologic outcomes has not been sufficiently explored. Previous retrospective analyses reported that LND at SRP was associated with lower cancer-specific mortality [2,3]. However, given the scarcity of this procedure, these studies suffered from a small sample size. In consequence, the oncologic effect of LND and LNI in SRP patients needs to be addressed in more detail and with a larger cohort size for more generalizable conclusions.

We hypothesized that LND has a protective effect on biochemical recurrence (BCR), cancer-specific survival (CSS), and overall survival (OS) in patients who underwent SRP for recurrent PCa. Moreover, we hypothesized that LNI might be associated with worse outcomes in BCR, CSS, and OS in SRP patients. We tested these hypotheses within a contemporary, large-scale multi-institutional database.

## 2. Material and Methods

### 2.1. Study Population

Patients that harbored histology-confirmed recurrent prostate cancer, between 2000 and 2021, at twelve high-volume centers were identified. The study was conducted after Institutional Review Board approval, and written informed consent was obtained from all patients. Salvage surgery was performed either with an open retropubic or robot-assisted laparoscopic approach as previously described for primary radical prostatectomy.

Exclusion criteria consisted of metastasis prior to SRP (*n* = 29), castration-resistant disease at the time of SRP (*n* = 26), or missing information on lymph node dissection status (*n* = 40). These selection criteria yielded 853 patients, who represent the focus of the current study.

### 2.2. Endpoints

BCR was defined as two consecutive PSA values ≥ 0.2 ng/mL after SRP. BCR was calculated as the time from SRP to the development of biochemical recurrence or last follow-up.

Overall survival (OS) was calculated as the time from SRP to death or last follow-up. Similarly, cancer-specific survival (CSS) was calculated as the time from SRP to death attributed to PCa or the last follow-up. Cancer-specific death was defined as death attributed to PCa diagnosis.

### 2.3. Statistical Analyses

Descriptive statistics included frequencies and proportions for categorical variables. Medians and interquartile ranges were reported for continuously coded variables. The chi-square tested the statistical significance of the proportions’ differences. The Mann–Whitney U test examined the statistical significance of medians’ differences respectively.

Kaplan–Meier analyses graphically depicted BCR-free survival, OS, and CSS rates. Two sets of multivariable Cox regression models were fitted to test the relationship between LNI, number of positive lymph nodes and number of removed lymph nodes, and the oncologic outcomes. Specifically, the first set of Cox regression models focused on BCR, and the second set of Cox regression models focused on death. The adjustment was made for the covariates: age at surgery, preoperative PSA value, pathologic tumor stage (pT2 vs. pT3a vs. ≥pT3b), surgical margin status, primary treatment type (radiotherapy vs. brachy vs. focal) and pathologic Gleason score (≤6 vs. 7 vs. ≥8). Models assessing death were additionally adjusted for the Charlson comorbidity index (CCI 0 vs. 1 vs. >1).

R software environment for statistical computing and graphics (version 4.2.2 for Mac, The R Foundation for Statistical Computing, Vienna, Austria) was used for all statistical analyses. All tests were two-sided with a level of significance set at *p <* 0.05.

## 3. Results

### 3.1. Descriptive Statistics

Overall, 853 patients were identified. Of those, 85% received LND at salvage RP (Table 1). The median follow-up was 27 months. Patients with LND were slightly older, and the mean age was 66 vs. 65 years in patients with LND vs. without LND (*p* = 0.04). The mean intraoperative blood loss was higher (527 vs. 277 mL, *p* < 0.001), mean operating time was significantly longer (191 vs. 147 min, *p* < 0.001), and patients had a mean longer hospital stay (5.4 vs. 3.3 days, *p* < 0.001) for LND vs. no LND. Most patients in the LND cohort (70%) were treated with an open retropubic approach, while in the cohort without LND, most (77%) were treated with a robotic-assisted approach. No differences in major complications (Clavien ≥ III) occurred between patients with LND vs. without LND (*p* = 0.5).

Of the 727 patients with LND during SRP, 21% (*n* = 151) harbored LNI (pN1). Of those, most (*n* = 108) harbored only 1–2 positive nodes (72%). The mean number of removed nodes was 13 in the entire LND cohort, which was significantly higher in pN1 patients vs. pN0 patients (16 vs. 13, *p* < 0.001). Patients with pN1 more frequently had positive surgical margins (39 vs. 24%, *p* < 0.001), pathologic stage ≥ T3b (55 vs. 26%, *p* < 0.001), and more frequently, a Gleason score ≥ 8 in the specimen (58 vs. 33%, *p* < 0.001) compared to pN0 patients (Supplementary Table S1).

### 3.2. Effect of LND and LNI on BCR after SRP

**Figure 1:**
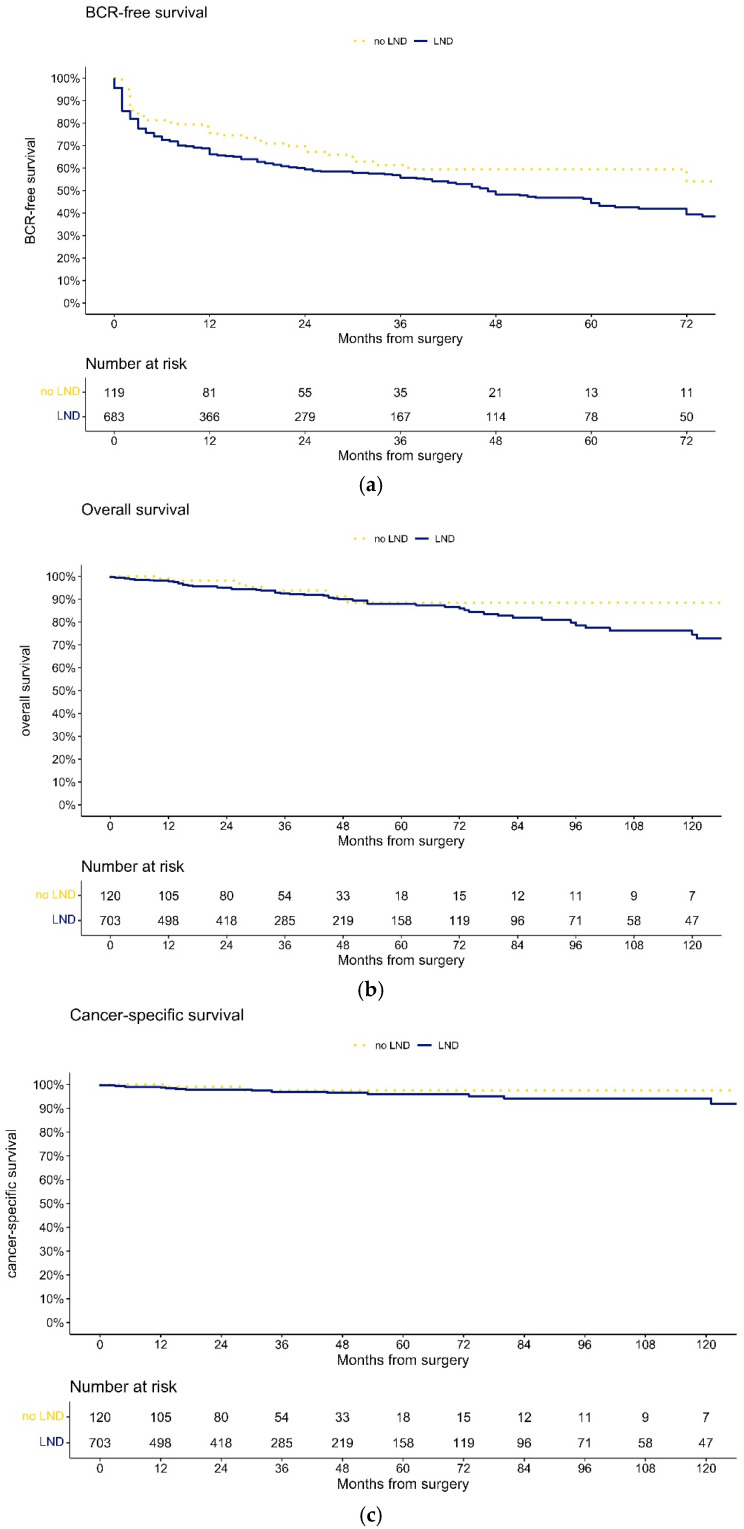
Kaplan–Meier plots depicting biochemical recurrence-free ((**a**); log-rank test: *p* = 0.1) and overall ((**b**); log-rank test: *p* = 0.4) and cancer-specific survival ((**c**); log-rank test: *p* = 0.4) for salvage RP patients according to lymph node dissection status (yellow dotted line no lymph node dissection performed, blue line lymph node dissection performed).

**Figure 2:**
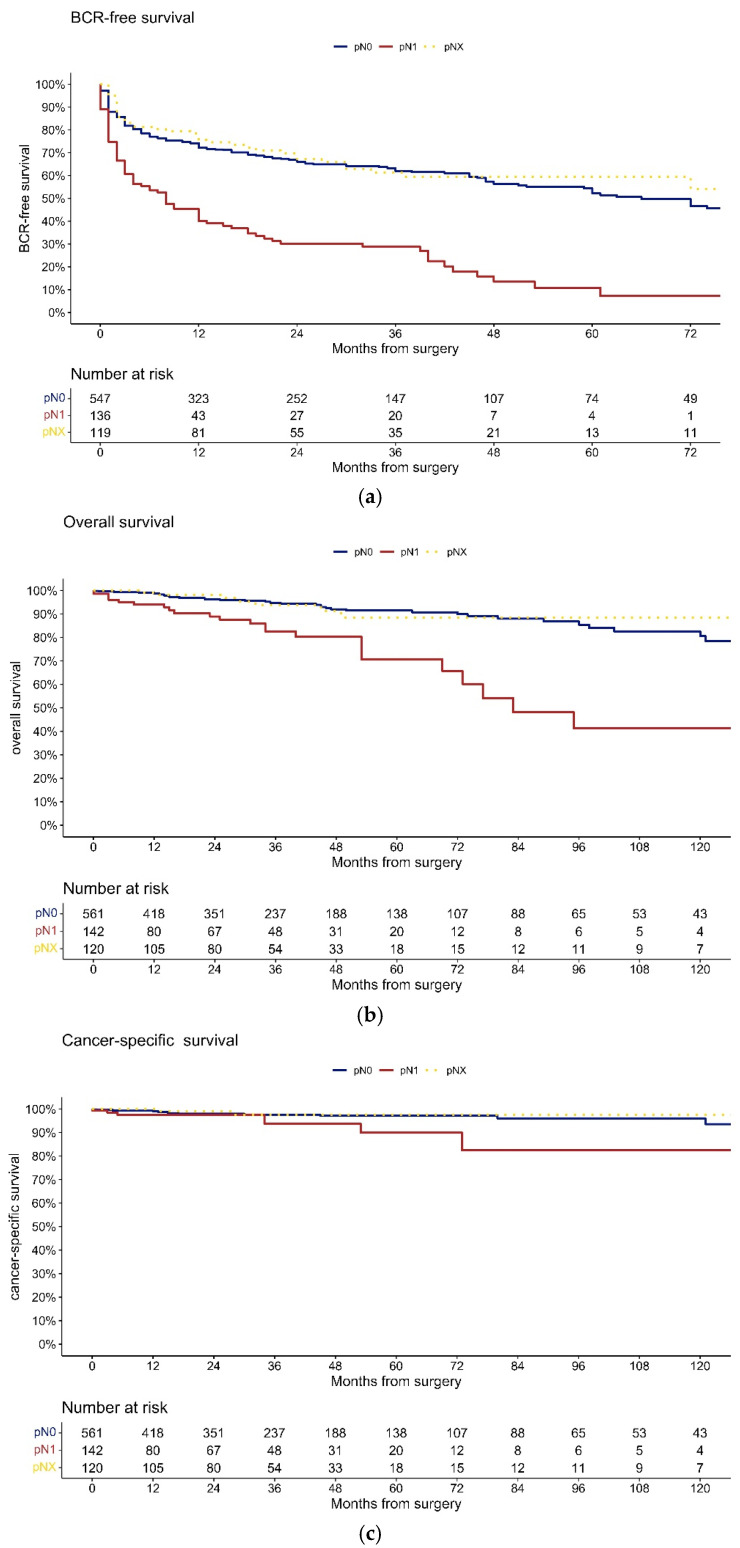
Kaplan–Meier plots depicting biochemical recurrence-free ((**a**); log-rank test: pN0 vs. pN1 *p* < 0.001, pN0 vs. pNx *p* = 0.7, pNx vs. pN1 *p* < 0.001) and overall ((**b**); log-rank test: pN0 vs. pN1 *p* < 0.001, pN0 vs. pNx *p* = 0.0, pNx vs. pN1 *p* = 0.001) and cancer-specific survival ((**c**); log-rank test: pN0 vs. pN1 *p* = 0.02, pN0 vs. pNx *p* = 0.6, pNx vs. pN1 *p* = 0.07) for salvage RP patients according to lymph node status (yellow dotted line: no lymph node dissection performed, pNx; blue line: lymph nodes negative, pN0; red line: lymph nodes positive, pN1).

At 72 months after SRP, BCR-free survival was 54% vs. 39% (Figure 1a, *p* = 0.1) for no LND vs. LND, respectively. When patients with LND were stratified according to lymph node status (Figure 2a), BCR-free survival at 72 months was 7.2% vs. 47% for pN1 vs. pN0 (*p* < 0.001).

In multivariable Cox models, pN1 was an independent predictor for BCR (hazard ratio (HR) 1.77, 95% confidence interval (95%-CI) 1.33–2.36, *p* < 0.001) (Table 2a). Additionally, in the same model, pathologic stage ≥ pT3b at SRP (HR 1.83, 95%-CI 1.35–2.47, *p* < 0.001), Gleason score ≥ 8 (HR 2.81, 95%-CI 1.44–5.49, *p* < 0.01) and positive surgical margins (HR 1.29, 95%-CI 1.01–1.65, *p* = 0.046) were also independent predictors for BCR.

Moreover, the number of positive lymph nodes (HR 1.13, 95%-CI 1.06–1.21, *p* < 0.001) was also an independent predictor for BCR (Table 2b). Conversely, the number of removed lymph nodes (Table 2c) was not associated with BCR (HR 0.99, 95%-CI 0.98–1.01, *p* = 0.4).

### 3.3. Effect of LND and LNI on Survival

At 120 months after SRP, OS rates were (Figure 1b) 89% vs. 75% (*p* = 0.4), and CSS rates (Figure 1c) were 98% vs. 94% (*p* = 0.4) for no LND vs. LND, respectively. When patients with LND were stratified according to lymph node status, OS (Figure 2b) and CSS (Figure 2c) rates at 120 months after SRP were 41% vs. 81%(*p* < 0.001) and 82% vs. 96% (*p* = 0.02) for pN1 vs. pN0, respectively.

In multivariable Cox models, pN1 was an independent predictor for death (HR 2.89, 95%-CI 1.62–5.13, *p* < 0.001) (Table 3a). Additionally, older age (HR 1.04, 95%-CI 1.01–1.09, *p* = 0.04) was also an independent predictor for death.

Moreover, the number of positive lymph nodes (HR 1.21, 95%-CI 1.12–1.31, *p* < 0.001) was also an independent predictor for death (Table 3b). Conversely, the number of removed lymph nodes (Table 3c) was not associated with death (HR 1.02, 95%-CI 0.99–1.04, *p* = 0.1).

Since only 22 patients died due to PCa, no multivariable adjustment could be performed for CSS.

## 4. Discussion

It is unknown to what extent LND and LNI affect oncologic outcomes in patients undergoing SRP. In the current study, we investigated the impact of LND and LNI on BCR, CSS, and OS after SRP. Our analysis revealed several novel and important findings.

First, within a multi-institutional database, we identified 853 patients who underwent SRP for recurrent prostate cancer. Our data represent the largest contemporary cohort of SRP patients. The second largest population of SRP patients (*n* = 427; 2004–2016) was identified within the Surveillance, Epidemiology, and End Results (SEER) database [3]. Other reports relied on single-institutional data ((*n* = 55; 2004–2008) [4]; (*n* = 55; 2007–2012) [5]), multi-institutional data ((*n* = 404; 1985–2009) [6]; (*n* = 96; 2001–2016) [7]; (*n* = 414; 200–2016) [8]) or the SEER database (*n* = 364; 1988–2010) [2]. These numbers underline the rarity of SRP. Concerns of higher complication rates of SRP compared to primary RP might explain the generally low case number of SRP. In consequence, the use of multi-institutional databases such as the current one is essential to provide generalizable observations for analyses of SRP patients.

Second, of the 727 patients with LND during SRP, 21% (*n* = 151) harbored LNI (pN1). This number is high considering that most of the previous studies on SRP patients reported lower LNI rates ranging from 6 to 22% [2,3,4,5,6,7,8]. It is of note that the LNI rate in SRP patients also depends on the number of removed LN. The mean number of removed LN in the current study was 13 in the entire LND cohort. Similarly, in recent primary RP cohorts, the median LN counts range from 14 to 16 [9,10]. Given the more difficult nature of the procedure, most recent SRP studies relied on removed LN numbers that were below those of the primary RP studies. For example, a median of six removed LN in SRP patients was reported in a population-based study (*n* = 427; 2004–2016) [3], and a median number of 11 (IQR 7–17) removed LN in SRP patients was reported in a multi-institutional study (*n* = 414; 200–2016) [8]. To the best of our knowledge, we are the first to provide a large-scale SRP population with a mean number of removed LN that is comparable to that of recent primary RP cohorts.

Third, important differences in baseline characteristics were identified for patients of various LN statuses. Specifically, comparing patients with pN1 vs. pN0, pN1 patients exhibited a higher rate of positive surgical margins (39 vs. 24%, *p* < 0.001), a higher rate of pathologic stage ≥ T3b (55 vs. 26%, *p* < 0.001) and a higher rate of Gleason score ≥ 8 in the specimen (58 vs. 33%, *p* < 0.001). Based on the variability of pathologic characteristics, multivariable adjustment for those differences is required in all analyses, where BCR and overall mortality represent an endpoint. Such methodology was used in the current study.

Fourth, omitting LND in SRP patients did not adversely affect the BCR-free survival rate. At 72 months after SRP, BCR-free survival was 54% vs. 39% (*p* = 0.1) for no LND vs. LND, respectively. Conversely, BCR-free survival at 72 months was significantly lower for pN1 vs. pN0 patients (7.2 vs. 47%, *p* < 0.001). Moreover, in multivariable Cox regression models, pN1 (HR 1.77, *p* < 0.001) as well as the number of positive LN (HR 1.13, *p* < 0.001) were independent predictors for BCR. However, the number of removed LN was not independently associated with BCR (*p* = 0.4).

Finally, omitting LND in SRP patients did not adversely affect OS and CSS. At 120 months after SRP, OS rates were 89 vs. 75% (*p* = 0.4), and CSS rates were 98 vs. 94% (*p* = 0.4) for no LND vs. LND, respectively. Conversely, OS and CSS rates at 120 months after SRP were significantly lower for pN1 vs. pN0 patients (OS: 41 vs. 81%, *p* < 0.001; CSS: 82 vs. 96%, *p* = 0.02). Moreover, in multivariable Cox regression models, pN1 (HR 2.89, *p* < 0.001) as well as the number of positive LN (HR 1.21, *p* < 0.001) were independent predictors for death. However, the number of removed LN was not independently associated with death (*p* = 0.1). The observations contradict previous findings, where LND vs. no LND in SRP patients was associated with a lower risk of cancer-specific mortality in two SEER-based studies [2,3]. Moreover, in one of these studies, the number of removed LN was also associated with lower cancer-specific mortality. However, this study relied on a median number of removed LN of only 6 (IQR 3–11), compared to 13 in the LND cohort of the current study. Another limitation of the SEER-based study was the missing comparison between pN1 vs. pN0 vs. pNX, which the current study provides with sufficient numbers of patients.

It is of note that in primary RP patients, no significant differences were shown between LND vs. no LND on oncologic outcomes in patients with D’Amico high- or intermediate-risk PCa [9]. Moreover, also in primary RP patients, the number of positive LN was independently associated with adverse oncologic outcomes, while the number of removed LN was not [11]. The current study demonstrates that these variables (LND vs. no LND; number of positive LN; number of removed LN) behave similarly in the salvage treatment setting.

Despite several new insights, our study is not devoid of limitations. First, it is limited by its retrospective nature. Second, omitting LND in some cases might be the result of a selection bias among surgeons. Third, in the multi-institutional database of the current study, an interobserver variability of pathologists for histological work-up of LN cannot be excluded. Fourth, different treatment modalities of radiation therapy and focal therapy and unavailable information on the use of concomitant androgen deprivation therapy, as well as information on the radiation therapy regime for primary PCa treatment, might have influenced our findings. Moreover, differences in imaging after and before SRP could also have accounted for limiting the homogeneity of our cohort. Specifically, PSMA-PET was not available at the time of the study and could have impacted treatment in those with positive lymph node metastases not identified on conventional imaging. Nevertheless, this is the first report assessing the effect of LND and LNI on various oncological outcomes (BCR, OS, and CSS) in a large contemporary cohort of SRP patients.

## 5. Conclusions

In SRP patients, LNI represents an independent poor prognostic factor. Moreover, LND at SRP represents a safe diagnostic tool. However, the oncologic benefit of LND in SRP remains debatable. These findings underline the need for a cautious LND indication in SRP patients as well as strict postoperative monitoring and, if necessary, adjuvant therapy of SRP patients with LNI.

## Figures and Tables

**Table 1:** Descriptive characteristics of 853 patients with recurrent prostate cancer that underwent salvage radical prostatectomy, stratified according to lymph node dissection performance.

Variable	No LND, *n* = 126 (15%) ^1^	LND, *n* = 727 (85%) ^1^	*p*-Value ^2^
PSA before SRP, ng/mL	6.6 (5.3)	6.4 (11.2)	0.8
Age at SRP, yrs	65 (6)	66 (7)	0.039
Lymph nodes removed	0 (0)	13 (9)	<0.001
Operating time, min	147 (64)	191 (72)	<0.001
Intraoperative bloodloss, mL	277 (186)	527 (498)	<0.001
Hospital stay, days	3.3 (3.8)	5.4 (3.5)	<0.001
Primary treatment type			<0.001
Radiotherapy	47 (37%)	435 (63%)	
Brachy	27 (21%)	144 (21%)	
Focal	52 (41%)	108 (16%)	
CCI			<0.001
0	47 (37%)	557 (77%)	
1	16 (13%)	52 (7.2%)	
>1	63 (50%)	118 (16%)	
Nerve sparing			<0.001
None	79 (63%)	367 (73%)	
Uni	23 (18%)	33 (6.5%)	
Bilateral	24 (19%)	104 (21%)	
Pathologic stage			0.5
≤pT2	66 (52%)	338 (47%)	
pT3a	25 (20%)	156 (21%)	
≥pT3b	35 (28%)	232 (32%)	
Major surgical complications (Clavien ≥ III)	8 (8.9%)	68 (11%)	0.5
Surgical approach			<0.001
ORP	29 (23%)	512 (70%)	
RARP	97 (77%)	215 (30%)	
Surgical margins			0.2
Negative	80 (66%)	527 (72%)	
Positive	41 (34%)	200 (28%)	
Biopsy Gleason score before SRP *			0.002
≤6	15 (14%)	122 (19%)	
7	70 (67%)	314 (48%)	
≥8	20 (19%)	212 (33%)	
Pathologic Gleason score *			0.019
≤6	7 (5.8%)	48 (6.9%)	
7	83 (69%)	385 (55%)	
≥8	31 (26%)	266 (38%)	

Abbreviations: CCI—Charlson comorbidity index; LND—lymph node dissection; ORP—open retropubic prostatectomy; PSA—prostatic-specific antigen; RARP—robotic-assisted laparoscopic prostatectomy; SD—standard deviation; SRP—salvage radical prostatectomy; * pathologic assessment might be affected by primary treatment modality. ^1^ Mean (SD); *n* (%); ^2^ Welch two-sample *t*-test; Pearson’s chi-square test.

**Table 2:** Multivariable Cox regression models predicting biochemical recurrence after SRP with (a) pathologic lymph node status, (b) number of positive lymph nodes, and (c) number of removed lymph nodes.

	(a)			(b)			(c)		
	HR	95%-CI	*p*-Value	HR	95%-CI	*p*-Value	HR	95%-CI	*p*-Value
PSA pre SRP	1.01	0.99–1.01	0.5	1.01	0.99–1.01	0.7	1.01	0.99–1.01	0.2
Age at SRP	1.01	0.99–1.03	0.3	1.01	0.99–1.03	0.2	1.01	0.99–1.03	0.3
Pathologic stage ≤ pT2 (reference)	1.00	-	-	1.00	-	-	1.00	-	-
Pathologic stage pT3a	1.38	0.99–1.91	0.1	1.32	0.93–1.88	0.1	1.45	1.02–2.05	0.04
Pathologic stage ≥ pT3b	1.83	1.35–2.47	<0.001	1.76	1.27–2.45	<0.001	2.04	1.48–2.80	<0.001
Pathologic Gleason score ≤ 6 (reference)	1.00	-	-	1.00	-	-	1.00	-	-
Pathologic Gleason score 7	1.82	0.95–3.48	0.1	1.52	0.79–2.94	0.2	1.72	0.89–3.32	0.1
Pathologic Gleason score ≥ 8	2.81	1.44–5.49	<0.01	2.72	1.39–5.31	<0.01	2.87	1.47–5.61	<0.01
Positive surgical margins	1.29	1.01–1.65	0.046	1.33	1.01–1.74	0.04	1.31	1.01–1.71	0.045
Primary treatment radiotherapy (reference)	1.00	-	-	1.00	-	-	1.00	-	-
Primary treatment brachytherapy	0.89	0.67–1.19	0.4	0.87	0.64–1.18	0.4			
Primary treatment focal therapy	0.94	0.69–1.29	0.7	0.80	0.55–1.16	0.2			
Pathologic lymph node status pN0 (reference)	1.00	-	-						
Pathologic lymph node status pNx	0.98	0.70–1.38	0.9						
Pathologic lymph node status pN1	1.77	1.33–2.36	<0.001						
Number of positive lymph nodes (continuously coded)				1.13	1.06–1.21	<0.001			
Number of removed lymph nodes (continuously coded)							0.99	0.98–1.01	0.4

Abbreviations: HR—hazard ratio; CI—confidence interval; PSA—prostatic-specific antigen; SRP—salvage radical prostatectomy.

**Table 3:** Multivariable Cox regression models predicting death after SRP with (a) pathologic lymph node status, (b) number of positive lymph nodes, and (c) number of removed lymph nodes.

	(a)			(b)			(c)		
	HR	95%-CI	*p*-Value	HR	95%-CI	*p*-Value	HR	95%-CI	*p*-Value
PSA pre SRP	1.01	0.99–1.03	0.7	1.01	0.98–1.03	0.9	1.01	0.98–1.03	0.6
Age at SRP	1.04	1.01–1.09	0.04	1.04	1.01–1.09	0.04	1.04	1.01–1.08	0.04
Charlson comorbidity score 0 (reference)	1.00	-	-	1.00	-	-	1.00	-	-
Charlson comorbidity score 1	0.49	0.19–1.25	0.1	0.55	0.21–1.43	0.2	0.50	0.19–1.28	0.1
Charlson comorbidity score > 1	1.34	0.77–2.32	0.3	1.05	0.56–1.95	0.9	1.09	0.59–2.02	0.8
Pathologic stage ≤ pT2 (reference)	1.00	-	-	1.00	-	-	1.00	-	-
Pathologic stage pT3a	0.90	0.41–1.95	0.8	0.76	0.32–1.81	0.5	0.85	0.37–1.95	0.7
Pathologic stage ≥ pT3b	1.41	0.75–2.64	0.3	1.18	0.61–2.27	0.6	1.54	0.80–2.97	0.2
Pathologic Gleason score ≤ 6 (reference)	1.00	-	-	1.00	-	-	1.00	-	-
Pathologic Gleason score 7	0.85	0.32–2.25	0.7	0.83	0.31–2.23	0.7	0.81	0.30–2.17	0.7
Pathologic Gleason score ≥ 8	1.16	0.42–3.21	0.8	1.42	0.51–3.95	0.5	1.16	0.42–3.23	0.8
Positive surgical margins	0.89	0.50–1.58	0.7	1.10	0.60–2.03	0.8	1.04	0.56–1.90	0.9
Primary treatment radiotherapy (reference)	1.00	-	-	1.00	-	-	1.00	-	-
Primary treatment brachytherapy	0.69	0.36–1.31	0.3	0.64	0.32–1.30	0.2	0.69	0.34–1.40	0.3
Primary treatment focal therapy	0.44	0.17–1.13	0.1	0.51	0.18–1.44	0.2	0.48	0.17–1.35	0.2
Pathologic lymph node status pN0 (reference)	1.00	-	-						
Pathologic lymph node status pNx	1.11	0.51–2.43	0.8						
Pathologic lymph node status pN1	2.89	1.62–5.13	<0.001						
Number of positive lymph nodes (continuously coded)				1.21	1.12–1.31	<0.001			
Number of removed lymph nodes (continuously coded)							1.02	0.99–1.04	0.1

Abbreviations: HR—hazard ratio; CI—confidence interval; PSA—prostatic-specific antigen; SRP—salvage radical prostatectomy.

## Data Availability

The datasets generated during and/or analyzed during the current study are available from the corresponding author on reasonable request.

## Supplementary Materials

The following supporting information can be downloaded at: https://www.mdpi.com/article/10.3390/cancers15123123/s1, Table S1: Descriptive characteristics of 727 patients with recurrent prostate cancer that underwent salvage radical prostatectomy and lymph node dissection, stratified according to lymph node negative (pN0) and positive (pN1).
