# Supplementary material for: Oncologic Outcomes of Lymph Node Dissection at Salvage Radical Prostatectomy
Source: Cancers (Basel). 2023 Jun 9;15(12):3123. doi: 10.3390/cancers15123123 (PMC10296518; doi:10.3390/cancers15123123)
Supplement: Supplementary file 1 [file cancers-15-03123-s001.zip › Supplementary Table S1 new.pdf]

Supplementary Table S1: Descriptive characteristics of 727 patients with recurrent prostate cancer that underwent salvage radical prostatectomy and lymph node dissection, stratified according to lymph node negative (pN0) and positive (pN1).

Variable	pN0, N = 576 (79%) [1]	pN1, N = 151 (21%) [1]	p-value [2]
**PSA before SRP, ng/ml**	5.4 (5.0)	10.5 (21.9)	0.005
**Age at SRP, yrs**	66 (7)	67 (7)	0.2
**Lymph nodes removed**	13 (9)	16 (10)	<0.001
**Original treatment type**			0.2
Radiotherapy	339 (62%)	96 (68%)	
Brachy	115 (21%)	29 (21%)	
Focal	92 (17%)	16 (11%)	
**Pathological stage**			<0.001
≤pT2	300 (52%)	38 (25%)	
pT3a	126 (22%)	30 (20%)	
≥pT3b	149 (26%)	83 (55%)	
**Surgical approach**			0.7
ORP	404 (70%)	108 (72%)	
RARP	172 (30%)	43 (28%)	
**Surgical margins**			<0.001
Negative	435 (76%)	92 (61%)	
Positive	141 (24%)	59 (39%)	
**Biopsy Gleason score before SRP ***			<0.001
≤6	106 (21%)	16 (12%)	
7	261 (51%)	53 (40%)	
≥8	149 (29%)	63 (48%)	
**Pathologic Gleason score ***			<0.001
≤6	46 (8.3%)	2 (1.4%)	
7	326 (59%)	59 (41%)	
≥8	183 (33%)	83 (58%)	

[1] Mean (SD); n (%)

[2] Welch Two Sample t-test; Pearson's Chi-square test

Abbreviations: ORP – open retropubic prostatectomy, PSA – prostatic specific antigen; RARP – robotic assisted laparoscopic prostatectomy; SD – standard deviation ;SRP – salvage radical prostatectomy.

* pathologic assessment might be affected by primary treatment modality.
